# A study on paracetamol cardiotoxicity

**DOI:** 10.1186/s40360-016-0073-x

**Published:** 2016-07-14

**Authors:** Udaya Ralapanawa, Kushalee Poornima Jayawickreme, Ekanayake Mudiyanselage Madhushanka Ekanayake, A. M. S. Dhammika Menike Dissanayake

**Affiliations:** Department of Medicine, University of Peradeniya, Peradeniya, Sri Lanka; Department of Pathology, Faculty of Medicine, University of Peradeniya, Peradeniya, Sri Lanka

## Abstract

**Background:**

Sri Lanka has a high suicide rate, with more than 40 % of poisoning admissions due to overdose of drugs with Paracetamol being the commonest. Data regarding cardiotoxicity to paracetamol is very minimal though hepatotoxicity following poisoning is well studied. Paracetamol cardiotoxicity has rarely been clinically significant and may have well been overlooked. The possibility that paracetamol overdose might be directly cardiotoxic has been the subject of a few reports. Unexplained deaths and electrocardiographic changes associated with paracetamol poisoning have also been reported in which cardiac origin cannot be clearly ruled out. Although some studies state that paracetamol poisoning has no direct cardiotoxic effect, electrocardiographic changes due to metabolic derangement of hepatotoxicity have been shown in certain studies. Thus, we conducted this study to assess in detail the cardiotoxic effect of paracetamol poisoning.

**Methods:**

This is a cross sectional descriptive study done on those with confirmed paracetamol poisoning. Serum paracetamol levels, Electrocardiogram, Echocardiogram, troponin I, and other basic investigations were done.

**Results:**

Paracetamol ingestion is more common among teenagers and the young population in Sri Lanka. Although several cases of paracetamol poisoning induced cardiotoxicity has been described in the past, this study demonstrated no electrocardiographic, echocardiographic or cardiac biomarkers changes of myocardial toxicity.

**Conclusion:**

Though literature review support cardiotoxicity following paracetamol poisoning, our study does not provide enough evidence for this. Continuous cardiac monitoring, serial troponin and echocaediogram assessment would be voluble adjunct in its management. Further experiments and research in this subject would be useful with a larger number of samples to further evaluate this important problem.

## Background

Sri Lanka has a very high suicide rate, mainly due to acute poisoning with pesticides. Since 1995 the suicide rate in Sri Lanka has declined to 23 per 100,000 population in 2006, and is attributed to a drop in case fatality due to the reduction in toxicity of accessible pesticides, and the implication of regulations to restrict the accessibility of toxic pesticides [1]. Ingestion of pesticides as a method of self-harm has become less frequent in recent years in Sri Lanka. In urban districts in Sri Lanka, more than 40 % of poisoning admissions were due to the overdose of drugs, medicaments and biological substances but this trend is not restricted to urban areas alone. However, though there is a shift of substances used in self-poisoning from pesticides to medicinal drugs, there is a steep rise in rates of self-poisoning in most districts, which is a serious issue [2]. Over the past few years paracetamol poisoning has shown a marked increase. It is the most widely used over-the -counter analgesic/antipyretic. The main reason for paracetamol to be the commonest drug responsible for self-poisoning is that it is readily available [3–6]. It was the commonest drug responsible for deliberate self-harm in the National Hospital Sri Lanka in 2003 [4, 5]. It was also the commonest drug responsible for self-poisoning in the United Kingdom, with 25 000 admissions from paracetamol being recorded in 2001 alone [7].

Paracetamol is predominantly metabolised to glucuronide and sulphate conjugates, which are excreted in the urine. Hepatotoxicity is related to the conversion of a small proportion of the ingested dose to N-acetyl-p-benzoquinoneimine. In therapeutic doses, N-acetyl-p-benzoquinoneimine is detoxified by conjugation with glutathione in the liver, but hepatic and renal damage may ensue once the protective intracellular glutathione stores are depleted. N-acetylcysteine and methionine replenish glutathione stores in the liver and kidney. Methionine is given orally whereas N-acetylcysteine is available in oral and intravenous form. There is also some evidence that N- acetylcysteine reduces mortality in paracetamol induced liver failure, although the drug acts via a different mechanism from glutathione repletion [8–13].

Though hepatotoxicity following paracetamol poisoning is well studied data regarding cardiotoxicity is very minimal. While general experience suggests that paracetamol induced cardiotoxicity has rarely been clinically significant, it may have been overlooked. The possibility that an overdose of paracetamol might be directly cardiotoxic has been the subject of a few reports [14–19]. (Table 1) However the true incidence is unknown as serial electrocardiograph (ECG) monitoring is not part of routine clinical management. Moreover, anxieties have been expressed regarding unexplained deaths associated with paracetamol poisoning, with some occurring within 24 h of ingestion [13]. It cannot be ruled out that a number of these may have been cardiac in origin [9]. Dysrhythmias and other ECG abnormalities, especially of the ST segment or T wave, frequently occur in comatose encephalopathic patients [4, 12], but ST/T wave changes in non-encephalopathic subjects have been reported [4, 9, 12–14]. Sudden unexplained death due to paracetamol overdose may occur early before acute hepatic failure is established, and even in the complete absence of histological evidence of hepatic necrosis [9, 14]. A direct toxic myocarditis has been postulated from autopsy evidence of myocardial necrosis being present particularly in the sub endocardium [9, 14, 15]. Cases of Toxic myocarditis, acute myocardial necrosis following paracetamol poisoning have been reported [9, 11, 14, 15]. The cause of the ECG abnormalities in encephalopathic patients is probably multifactorial and at least partly related to severe metabolic changes. However, these cannot be implicated in patients with less hepatic damage or in sudden death early in the course of paracetamol poisoning [4, 9]. Because of this *Armour* et al. suggested an ECG to be taken on admission and checked daily if the overdose is significant [9] They also further stated that if ST/T wave abnormalities or a dysrhythmia are present, the treatment with a standard acetylcysteine infusion should be considered, probably irrespective of the plasma concentration of paracetamol or time laps from ingestion [9]. Thum and Borlak showed the presence of metabolic enzyme(Cytochrome p450s) in the heart and suggested the possibility of metabolic activation of paracetamol [16]. Also literature review showed a very minimal amount of research had been carried out to determine paracetamol cardio toxicity which seems to have been overlooked.

**Table 1:** Evidence of cardiotoxicity in paracetamol overdose

Evidence of cardiotoxicity in Paracetamol overdose	References
Toxic myocarditis	Reference 9,14,15,29,43
Acute myocardial necrosis	Reference 9,11,15,16,43
Subendocardial necrosis	Reference 9,14,15
Subendocardial haemorrhage	Reference 8
Patchy myocardial necrosis in left ventricular wall	Reference 15,32
Transient interventricular septal thickenning	Reference 15
Dysrhythmias	Reference 9,14,15,29
ST/T wave inversions	Reference 9,15,17,29,42
Flatten T waves	Reference 14,17,43
ST elevation	Reference 9,15,29,43
Sinus tachycardia	Reference 9,15
Ventricular tachycardia	Reference 9,15
Multifocal ventricular ectopics	Reference 19
Cardiac asystole	Reference 15
Dilated left ventricle	Reference 9,15,43
Elevated Troponin	Reference 15,29
Acute severe left ventricular failure	Reference 29,32
Congestive heart failure	Reference 32
Cardiomyopathy	Reference 15,26

The above evidence would justify paracetamol cardiotoxicity being studied in detail amongst our patients. We carried out a study on patients presenting to the Toxicology Unit of the Teaching Hospital Peradeniya. We selected this unit of the Teaching Hospital Peradeniya as all acute poisoning patients are admitted here.

## Methods

The objective of our study was to assess the demographic and clinical patterns of paracetamol poisoning induced cardiotoxicity in the Sri Lankan population. The study population included all patients with a history of Paracetamol poisoning presenting to the toxicology unit of the Teaching Hospital Peradeniya over a period of one year. All patients who were clinically diagnosed to have paracetamol poisoning after detailed history at the time of presentation were included in this study. Those patients with multiple drug poisoning and those who refused to participate were excluded from the study. Ethical clearance was obtained from the Ethical Review Committee of the Faculty of Medicine, University of Peradeniya, and informed written consent was obtained from all subjects prior to data collection.

All patients presenting with paracetamol poisoning to the Toxicology Unit of the Teaching Hospital Peradeniya were assessed by detailed history and examination by investigators recruited to the study. The patients who were confirmed to have a paracetamol poisoning were selected for the study. The toxic dose of paracetamol for adults is considered to be either a single dose of 7.5–10 g, which is equivalent to 20 tablets of 500 mg each, or repeated sub-therapeutic doses exceeding 100 mg/kg/day. As for healthy children aged between 1 and 6 years the toxic dose is considered to be between 150 and 200 mg/kg. This toxic dose is defined by the occurrence of hepatotoxicity which is the main toxic effect of paracetamol poisoning, and the cardiotoxic dose has not yet been determined. All the subjects of this study were above the age of 15, therefore all subjects who had consumed more than 20 paracetamol tablets were considered to have paracetamol poisoning.

**Fig. 1:**
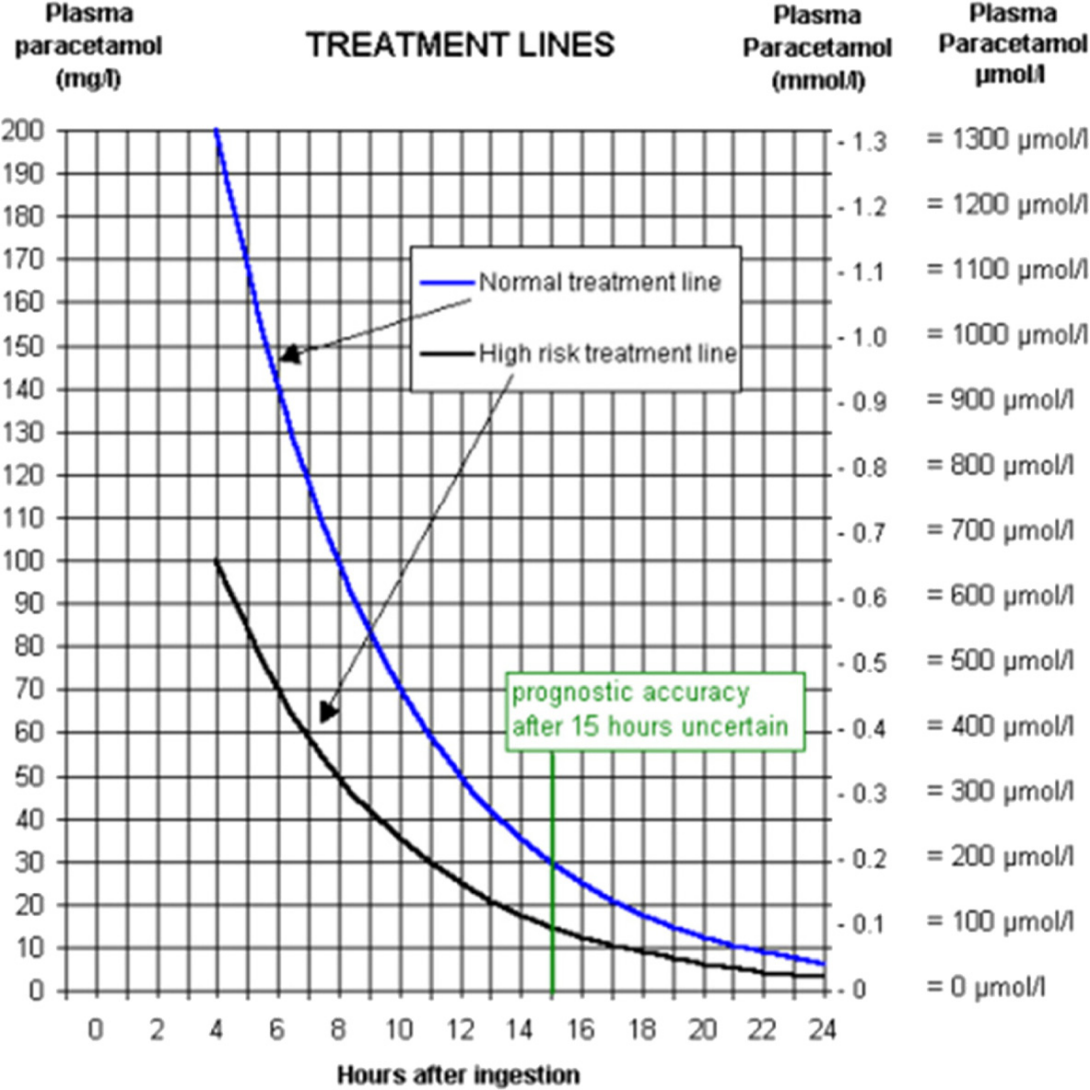
Paracetamol overdose treatment nomogram

All the study subjects underwent routine hematological and biochemical investigations on admission, followed by 4 h blood paracetamol level. In case of unknown time of ingestion random blood paracetamol level was measured. Blood paracetamol concentration level against the time since ingestion was plotted on the Rumak-Mathew normogram (Fig. 1) to assess paracetamol toxicity.

Liver functions and renal functions were assessed daily over the next few days. Daily 12 leads ECG was taken, and was interpreted by a Consultant Physician specialized in Cardiology. Serum Troponin I level was measured at 4 h after ingestion of paracetamol in patients who were found to have detectable serum paracetamol levels. In addition basic demographic data was also obtained. Data was collected using a comprehensive data collection sheet and was analyzed using the SPSS (Statistical package for social sciences) 20.0 software.

## Results

### Basic socio-demographic data

Studied sample included 68 patients who are ingested 20 or more Paracetamol tablets, with a mean age of 21.38 +/- 5.11 (range 15–36). This included 26(38.2 %) male patients and 42(61.8 %) females (Table 2) and reason for Paracetamol ingestion is given in Table 3. Clinical findings on admission were recorded and is shown in Table 4.

**Table 2:** Age and sex Distribution

Age Group	Number Total = 68	Sex	
		Male(n 26)	Female(n 42)
<18	22(32.4 %)	8	14
18-24	31(45.6 %)	11	20
>24	15(22.0 %)	7	8

**Table 3:** Reason for Paracetamol ingestion

Reason for Paracetamol ingestion	Number of patients	Percentages (%)
Impulsive action	24	35.3
Deliberate self-harm	14	20.6
Suicidal	30	44.1

**Table 4:** Clinical data on admission

Clinical findings	Number of patients	Percentages %
Nausea	55	80.9
Vomiting	48	70.6
Chest Pain	20	29.4
RUQ pain	15	22.1
Jaundice	2	2.9
Confusion	1	1.5
Easy Bleeding	0	0

Out of 68 patients, 58(85.3 %) were Sinhalese, 9(13.2 %) were Muslims and 1(1.5 %) patient was Christian; 16(23.5 %) patients were regular smokers and 18(26.5 %) were regular alcohol users.

Among the Studied population, 44(64.7 %) only completed their primary education (Ordinary level), 21(30.9 %) educated up to Advanced level and only 3(4.4 %) completed tertiary education.

Out of 68, 47(69.12 %) answered the source of paracetamol as Home and 21(30.88 %) patients were taken Paracetamol over the counter.

All the patients had ingested paracetamol tablets and the mean number of paracetamol tablets was 37+/- 18.36 (Range20–96) (19 g); 2(2.9 %) of them ingested alcohol with paracetamol and 3(4.4 %) ingested cholropheniramine with paracetamol.

Of the 68 subject, 27 (39.70 %) had detectable paractamol levels in serum, and 21 (30.88 %) had Alanine Transaminase levels (ALT) ≥ 1000 IU/L.

Out of studied sample (68), 9 (13.2 %) had a past history of Depression, 11(16.2 %) had a chronic illness, and only 10(14.7 %) had a history of previous suicidal attempts. Eight(11.8 %) had family history of suicide and 6(9 %) had family history of depression.

ECG and Echocardiography were done in all patients. Approximately, 20.59 % had bradycardia while 2.94 % had tachycardia. Majority(76.47 %) had normal heart rate. Fourteen patients(20.59 %) had first degree heart block. The duration of QRS was normal in all the patients. Small Q waves were detected in 13.24 % patients and they were not pathological. ST segment depression was observed in 2.94 % patients and was less than 1 mm and non significant.10.29 % patients had T wave inversions in inferior leads and were considered normal (Table 5). Standard treatment was given to all patients on admission and during the stay in the hospital (Table 6).

**Table 5:** Electrocardiogram findings of the sample

	Heart Rate			PR interval(Sec)			QRS duration(Sec)		Q wave		ST depresion		T inversion	
	<60	60-100	>100	<0.12	0.12-0.20	>0.2	<0.12	>0.12	Present	Absent	Present	Absent	Present	Absent
Number	14(20.59 %)	52(76.47 %)	2(2.94 %)	0	54(79.41 %)	14(20.59 %)	68(100 %)	0	9(13.24 %)	59(86.76 %)	2(2.94 %)	66(97.09 %)	7(10.29 %)	61(89.71 %)

**Table 6:** Treatment modalities

Modality	Number	Percentages
Gastric Lavage	23	39
Activated Charcoal	29	42.1
NAC	47	81
Methionine	11	18.6

Echocardiogram was done on all the patients and did not reveal any significant abnormality. All of them had normal ejection fraction and there were no any regional wall motion abnormalities. There were no hypokinetic or akinetic segments.

Cardiac Biomarker Troponin I was done only in patients who had detectable serum paracetamol levels (27 Patients). Surprisingly Troponin I was negative in all of them.

The average time taken for hospital admission following paracetamol ingestion was 3.25 ± 2.27 h.

## Discussion

Paracetamol is the most commonly encountered drug leading to toxicity due to overdose and can lead to severe complications and even result in death. The commonest organ system involved in toxicity of paracetamol is the liver.(H,I) A few previous studies have reported cases of paracetamol toxicity affecting organs other than the liver, including the heart, which was assessed in detail by this study [10, 15, 17–19].

Currently there are a few hypothesized mechanisms for the occurrence of the previous reported cases of cardiotoxicity due to paracetamol overdose, which could either be a direct or indirect effect. A few studies have shown the Acetaminophen moiety to have a direct toxic effect on the heart [9, 14, 20–23]. One hypothesis for the direct toxic effect on the heart suggests that myocardial injury occurs due to a similar mechanism which causes hepatic damage, with paracetamol partly converted to a toxic metabolite, N-acetyl-p-benzoquinonimine, which is normally inactivated by reduction with glutathione and acts as a direct toxin on the myocardium [9]. Paracetamol has shown to bind covalently to proteins in both the liver and cardiac tissue which results in the alteration of protein structure and function, potentially precipitating cytokine release and tissue injury [22, 23]. Another hypothesis is one resulting in ischemia due to paracetamol, which can be due to the depletion of sulfydryl groups. Acetaminophen can deplete sulfydryl groups, which interferes with nitric oxide production, which results in coronary ischaemia [9, 20]. This sulfydryl deficit also may interfere with endothelium derived vascular relaxing factor, which leads to functional coronary ischemia [9, 20].

The most commonly postulated hypothesis for the mechanism for cardiotoxicty in paracetamol poisoning is metabolic derangements such as hyperkalemia, metabolic acidosis and increase in serum fatty acid levels, resulting by hepatic failure, which could lead to arrythmias [9, 12]. Previously reported cases of paracetamol poisoning with cardiac tissue damage has almost always been associated with hepatotoxicity. *Lesna* et al. stated that there have been no cases of cardiac arrest so far in the absence of hepatotoxicity, which further justifies the hypothesis which states that cardiac tissue damage in paracetamol poisoning occurs following metabolic derangements due to hepatotoxicity [18, 24–27]. Similarly, in this study 30.88 % had ALT ≥ 1000 IU/L indicating hepatic tissue injury, but all had negative Troponin I, echocardiogram and ECG, indicating the absence of cardiac tissue injury in any of the subjects. A case of myocardial damage following acute paracetamol poisoning by *Ohtani* et al. reported heart failure to occur 2 weeks following hepatic injury, and had return of hepatic function tests to normal and had no elevated levels of serum paracetamol by that time, which shows that though there may be no features of cardiac tissue injury or impairment at the time there is hepatic damage, but cardiac toxicity could occur at a later stage even in the absence of features of hepatic tissue injury at that stage [28–32]. *Mi Jin* et al. in their experiment have demonstrated that paracetamol induced cytotoxicity and altered gene expression in cultures cardiomyocytes of H9C2 cells [23]. This study was plan to identify the alterations in gene expression in cultured cardiomyocytes;H9C2 cells, which are rat cardiomyocytes, were treated with paracetamol, to evaluate the possible toxic mechanism in the heart. During this study authors have demonstrated some of the DNA damage and upregulation of genes including those associated with oxidative stress, DNA damage and apoptosis [23]. During this study to examine toxic effects of paracetamol,H9C2 rat cardiomyocytes were incubated with different concentratins of paracetamol and viability was determined at different time intervals and showed H9C2 cells viability was decreased by treatment with paracetamol in a time- and dose-dependant manner [16]. Another laboratory study done by *Inoue* et al. to study cardiotoxicity and hepatotoxicity of different compounds using neonatal rat heart cells and rat hepatocytes confirmed that paracetamol was cardiotoxic in significant overdose [33].

Previous studies have shown that cardiotoxicity following paracetamol poisoning may be either immediate or delayed [20, 21], similar to the case reported by *Ohtani* et al. which showed a delay in onset of cardiac involvement [28]. The average time taken for hospital admission following paracetamol ingestion in this study was 3.25 ± 2.27 h. Probably if these patients were followed up weeks later, some of them may have developed cardiac toxicity and showed cardiac tissue damage by elevated troponin I, or positive echocardiogram or ECG changes. Since the cardiotoxicity due to paracetamol poisoning is believed to be either immediate or delayed, it is of paramount importance to administer N-acetylcysteine (NAC) as early as possible because it may be beneficial in reducing the functional coronary ischaemia as well as the direct toxic effects, irrespective of the time of ingestion [34–40]. Since a majority of 81 % of subjects were treated with NAC, and since the time taken for hospital admission following paracetamol ingestion was short, effective treatment with NAC would’ve been protective against the development of cardiotoxicity in this sample. Gastric aspiration or lavage should be carried out, or emesis induced within 4 h of ingestion of 100 mg/kg of paracetamol to reduce absorption, and this has been carried out in 39 % of patients. Activated charcoal and cholestyramine reduce absorption of paracetamol if given within 1 h of ingestion [24–26]. 42.1 % of the patients underwent treatment with charcoal, but since it was done after 1 h of ingestion, the protective effect from this measure would have been minimal or absent. However, other effective measures for the treatment of paracetamol poisoning may be the reason for cardiotoxicity not to develop in these patients.

In contrast to the believed toxic potential of paracetamol on the heart, literature has also demonstrated paracetamol to have a cardioprotective potential in ischaemia reperfusion syndrome. At concentrations of 45–50 mg/ml, well below the clinically toxic dose of 300 mg/ml, paracetamol can reduce tissue injury by preventing the opening of the mitochondrial permeability transition pore and reducing apoptosis [27, 28]. This dose dependent cardioprotective effect has a different mechanism from that of the cardiotoxic effect [41, 42]. However, the mean amount of paracetamol ingested by this study sample was 19 g, accounting to about 315 mg/kg, indicating that this cardioprotective effect of paracetamol is quite unlikely in this sample according to the facts stated in literature.

In patients with severe paracetamol poisoning, the ECG often reveals minor nonspecific ST changes and T wave flattening with U waves during the first 48 h, which is likely to be related to hyperkalaemia, but not due to direct cardiotoxicity [29]. Nonspecific ECG changes and elevated serum creatine kinase has been reported without clinical evidence of toxic myocarditis [30, 31]. A post mortem analysis of 20 fatal cases of paracetamol poisoning showed that there was no evidence of cardiomyopathy. Some degree of focal necrotizing myopathy has been found to occur in patients with fatal massive hepatic necrosis of diverse etiology [32, 33]. Serious cardiovascular complications in paracetamol overdose are not heard of in the absence of hepatic failure, and cardiac abnormalities reported in certain fatal cases are due to the profound metabolic disturbances of acute liver failure rather than paracetamol itself.

In contrast, there have been a few very rare cases of paracetamol overdose reported with abnormal ECG patterns in the absence of hepatotoxicity, metabolic derangement or encephalopathy [32, 43]. A similar case of a 29 year old man with significant paracetamol overdose was found to have an abnormal ECG in the absence of hepatic encephalopathy [9].

Approximately 35.3 % ingested paracetamol overdose by impulsive action, 20.6 % for deliberate self-harm, and 44.1 % for suicide. 11.8 % had a family history of suicide and 9 % had a family history of depression. Considering these facts, there is also a possibility that a certain number of patients may have mentioned an exaggerated number of paracetamol tablets ingested, and some may also not be very sure of the time and details of ingestion of the overdose. There is also the possibility that paracetamol tablets may have a more diluted concentration of the drug in its composition with a hindrance of quality, resulting in a lower level of serum paracetamol than expected compared to the number of tablets ingested.

## Conclusion

Cardiotoxicity is not a common presentation in paracetamol poisoning. It further justifies certain previous studies which state that if at all any cardiac tissue damage only occurs due to metabolic derangements following hepatotoxicity.

## Abbreviations

ALT, Alanine Transaminase levels; ECG, Electrocardiograph; NAC, N-acetylcysteine

## References

[1] Gunnell D, Fernando R, Hewagama M, Priyangika WDD, Konradsen F, Eddleston M (2007). The impact of pesticide regulations on suicide in Sri Lanka. Int J Epidemiol.

[2] Hanwella R, Senanayake SM, de Silva VA (2012). Geographical variation in admissions due to poisoning in Sri Lanka: a time series analysis. Ceylon Med J.

[3] Hawton K, Ware C, Mistry H (1995). Why patients choose paracetamol for self poisoning and their knowledge of its dangers. BMJ : British Medical Journal.

[4] Galappatthy P, Dawson A, Fernando R (2006). Management of paracetamolovedose. Sri Lanka Prescriber.

[5] Rajapakse T, Griffiths KM, Christensen H (2013). Characteristics of non-fatal self-poisoning in Sri Lanka:a systematic review. BMC Public Health.

[6] Eddleston M, Karunaratne A, Weerakoon M, Kumarasinghe S, Rezvi Sheriff MH, Gunnell D (2006). Choce of poison for intentional self-poisoning in rural Sri Lanka. Clin Toxicol(Phila).

[7] Morgan O, Griffiths C, Majeed A (2005). Impact of paracetamol pack size restrictions on poisoning from paracetamol in England and Wales: An observational study. J Public Health.

[8] Routledge P, Allister Vale J, Nicholas Bateman D (1998). BMJ.

[9] Armour A, Slater SD (1993). Paracetamolcardiotoxicity. Postgrad Med J.

[10] Isbister GK, Downes F, Whyte M (2003). Regular medication and paracetamol overdose. Aliment Pharmac of Ther.

[11] Tenenbein M (2004). Acetamenophen:the 150mg/kg myth. A Toxicol Clin Toxicol.

[12] Anderson BD, Shepherd JG, Klein-Schwartz W (1998). Outcome of acetaminophen overdose. J Pediatr.

[13] Hoffman C, Gibel L (2005). Acetomenophen overdose. Nursing.

[14] Will EJ, Tomkins AM (1971). Acute myocardial necrosis in paracetamol poisoning. Br Med J.

[15] Wakeel RA, Davies HT, Williams JD (1987). Toxic myocarditis in paracetamol poisoning. Br Med J.

[16] Weston MJ, Talbot IC, Howorth PJN, Mant AK, Capildeo R, Williams R (1976). Frequency of arrhythmias and other cardiac abnormalities in fulminant hepatic failure. Br Heart J.

[17] Mehrpour O, Afshari R, Delshard P, Jalazaden M, Khodashenas M (2011). Cardiotoxicity due to paracetamol overdose-A case study and review of the literature. Indian Journal of Forensic Medicine and Toxicology.

[18] Smilkstein MJ (1996). APAP-induced heart injury?may be yes.may be no.next question?. J Toxicol, Clin Toxicol.

[19] Brent J (1996). New ways of looking at an old molecule. J Toxicol, Clin Toxicol.

[20] Lim AY, Segarra I, Chakravarthi S, Akram S, Judson JP (2010). Histology and biochemistry analysis of the interaction between sunitinib and paracetamol in mice. BMC Pharmacol.

[21] Jacob S, Cherian P, Preusz C, Kovacs R (2008). Heartbreaking case of Acetaminophen poisoning. Circulation.

[22] Sanerkin NG (1971). Acute myocardial necrosis in par-acetamol poisoning. Br Med J.

[23] Jin SM, Park K (2012). Acetaminophen induced cytotoxicity and altered gene expression in cultured cardiomyocytes of H9C2 cells. Environmental Health and Toxicology.

[24] McKay C (2011). Acetamenophen: hidden complexities of a simple overdose. MLO Med Lab Obs.

[25] Hamwi I, Picksak G, Stichtenoth DO (2008). Accidental acetaminophen overdose. Med Monatsschr Pharm.

[26] Pimstone BL, Uys CJ (1968). Liver necrosis and myocardiopathy following paracetamoloverdosage. S Afr Med J.

[27] Lesna M, Watson AJ, Douglas A, Hamlyn AN, James O, Wardle EN, Chenery R, Fisher C, McLean AEM. Toxicity of paracetamol. The Lancet 307(7952):191

[28] Ohtani N, Matsuzaki M, Anno Y, Ogawa H, Matsuda Y, Kusukawa R (1989). A case of myocardial damage following acute paracetamol poisoning. Japanese Ciculation Journal.

[29] Jones AL, Prescott LF (1997). Unusual complications of paracetamol poisoning. Q J Med.

[30] Lip GYH, Vale JA (1996). Does acetaminophen damage the heart?. Clin Toxicol.

[31] Lacour S, Gautier J-C, Pallardy M, Roberts R (2005). Cytokines as potential biomarkers of liver toxicity. Cancer Biomark.

[32] Weston MJ, Williams R (1976). Letter:Paracetamol and the heart. Lancet.

[33] Inoue T, Tanaka K, Mishima M, Watanabe K (2008). Predictive *in vitro* cardiotoxicity and hepatotoxicity screening system using neonatal rat heart cells and rat hepatocytes. AATEX.

[34] Dordoni B, Willson RA, Thompson RPH, Williams R (1973). Reduction of absorption of paracetamol by activated charcoal and cholestyramine: a possible therapeutic measure. Br Med J.

[35] Levy G, Houston JB (1976). Effect of activated charcoal on acetaminophen absorption. Pediatrics.

[36] Prescott LF, Critchley JAJH, Balali-Mood M, Pentland B (1981). Effects of microsomal enzyme induction on paracetamol metabolism in man. Br J Clin Pharmacol.

[37] Hamlyn AN, Douglas AP, James O (1978). The spectrum of paracetamol (acetaminophen) overdose: clinical and epidemiological studies. Postgrad Med J.

[38] Maclean D, Peters TJ, Brown RAG, McCathie M, Baines GF, Robertson PGE (1968). Treatment of acute paracetamol poisoning. Lancet.

[39] Dixon MF (1976). Paracetamol hepatotoxicity. Lancet.

[40] Ojeda VJ, Shilkin KB, Wright EA, Williams R (1982). Massive hepatic necrosis and focal necrotising myopathy. Lancet.

[41] Hadzimichalis NM, Baliga SS, Golfetti R, Jaques KM, Firestein BL, Merrill GF (2007). Acetaminophen-mediated cardioprotection via inhibition of the mitochondrial permeability transition poreinduced apoptotic pathway. Am J Physiol Heart Circ Physiol.

[42] Merrill G, McConnell P, Vandyke K, Powell S (2001). Coronary and myocardial effects of acetaminophen: Protection during ischemiareperfusion. Am J Physiol Heart Circ Physiol.

[43] Contractor H, Gauge V, Nabi S, Titu H, Arya S, Naqvi N (2011). ST segment elevation secondary to paracetamol overdose. Ther Adv Cardiovasc Dis.

